# Unveiling triple vulnerability among Mozambican female sex workers—Stigma, physical violence and sexual violence

**DOI:** 10.1371/journal.pone.0312550

**Published:** 2025-02-21

**Authors:** Naira Luiz, Rachid Muleia, Ana Abecasis, Auria Banze, Denise Langa, Cynthia Semá Baltazar

**Affiliations:** 1 Instituto Nacional de Saúde, Maputo, Mozambique; 2 Instituto de Higiene e Medicina Tropical, Universidade Nova de Lisboa, Lisboa, Portugal; Johns Hopkins University Bloomberg School of Public Health, UNITED STATES OF AMERICA

## Abstract

**Background:**

In the shadows of Mozambique’s urban landscape, an invisible struggle unfolds among its most vulnerable: Female Sex Workers (FSWSs). FSWs bear a disproportionate burden of violence as a consequence of the stigma surrounding their profession, as both stigma and violence create significant barriers to the progress of HIV elimination within this group by limiting their access to prevention and treatment services, discourages them from seeking help, while violence itself increases vulnerability to HIV. This study examines the patterns of stigma, physical and sexual violence, and HIV among FSWs.

**Methodology:**

A secondary analysis was performed using data from a cross-sectional Bio-Behavioral Survey (BBS) conducted among FSW ≥15 and old, implemented between 2019–2020 in five urban areas. Respondent-driven sampling (RDS) was utilized to recruit participants. Aggregate weighted estimates were calculated for self-reported stigma, physical, and sexual violence. Associations between variables were assessed using chi-squared tests, and multivariate logistic regression was employed to identify factors associated with stigma, physical violence, and sexual violence.

**Results:**

Among 2,567 FSWs surveyed, 24.7% reported experiencing stigma, while 52.3% and 37.9% reported physical and sexual violence, respectively, in the six months preceding the survey. The likelihood of experiencing stigma was over six times higher for FSWs who engaged with more than 7 clients (AOR = 6.1; p <0.001). Drug use was associated with a twofold increase in the odds of physical violence (AOR = 2.3; p <0.001) and a nearly threefold increase in the odds of sexual violence (AOR = 2.7; p <0.001). HIV-positive FSWs were at increased risk for both physical violence (AOR = 1.2; p = 0.006) and sexual violence (AOR = 1.2; p = 0.031).

**Conclusion:**

This study highlights the substantial burden of stigma and violence among FSWs in Mozambique’s urban areas. The findings underscore the urgent need for targeted interventions to reduce stigma, prevent violence, and protect the rights of FSWs. Addressing these issues is essential for achieving the goals of HIV prevention and treatment in this vulnerable population.

## Introduction

Sex work is often defined as a consented exchange of sexual favors for money, goods or other benefits [1], and UNAIDS defines sex workers as women, men and transgender individuals who receive money or other remuneration in exchange for sexual favors weather regularly or occasionally [2]. Female Sex Workers (FSWs), for instance, represent a significant portion of this workforce and face unique challenges and societal perceptions. Their experiences often differ from those of their male and transgender counterparts due to various factors, such as societal attitudes, types of services offered, and specific vulnerabilities, including a higher risk of gender-based violence [1].

Throughout different communities, sex work can assume different forms and has different legal interpretations. In Africa, there are two contexts, one where sex work and related activities are considered illegal, and the second where sex work itself is not criminalized but solicitation of sex work in public is illegal [3]. The criminalization of sex work and the absence of policies to protect this group not only contribute to the violation of their rights but also increase the stigma and discrimination toward this population because it increases their vulnerability, which automatically affects their safety and health, as they are at high risk of contracting HIV [2, 4]. Evidence shows that a sex worker from a country where sex work is criminalized has a 7 times greater chance of having HIV when compared to a country where sex work is partially legalized [2].

Stigma plays an important role in discrimination, fueling social inequalities and causing victims to experience feelings of exclusion and depreciation [5]. The stigma surrounding FSW throughout the world comes from the negative perception that they are immoral and disease vectors [6, 7], causing them to be rejected by society [8]. Additionally, societal norms and cultural attitudes that view sex work as a deviation from traditional gender roles further exacerbate this stigma [9]. Such misconceptions not only marginalize FSWs but also create barriers to their access to healthcare, legal protection, and social acceptance, significantly impacting their quality of life and safety [9, 10]. This stigmatization often challenges the efforts being made to reduce the spread of HIV epidemics by increasing HIV status awareness [9–11].

Another critical factor that contributes to the vulnerability of FSW to HIV is physical and sexual violence, which often manifests as a direct result of the stigma directed toward this group, impacting the safety, health and quality of life of these women [12]. As a criminalized, stigmatized and marginalized group, FSW are more likely to experience violence while less likely to receive support when needed, either from the police or healthcare providers, hindering them from reporting incidents and allowing the violence cycle to repeat itself due to the impunity of the perpetrator [13].

Episodes of physical violence usually take place during the negotiations of the encounter, such as duration and condom use, and the likelihood of these negotiations escalating into violence is notably higher in situations involving drug and alcohol use [14]. Violence increases the vulnerability of FSW to HIV infection, as it hinders their access to information and services that can help protect them from HIV, impacting the progress of the ending HIV/AIDS epidemic as a public health threat by 2030 (8). Additionally, experiencing violence reduces the likelihood of FSWs seeking essential healthcare services following assaults, including post rape prophylaxis, HIV testing, and treatment for trauma [10, 14].

Despite Mozambique´s ranking among the nations with the highest HIV prevalence and new infections globally [15], there is a notable lack of research addressing the intersection of violence, stigma, and their impact on the HIV epidemic. A study among FSW of three provinces of Mozambique revealed that 11.1% out of the total participants were victims of physical violence in the six months prior to the survey [14], and another study among FSW showed that 70% of the respondents reported experiencing violence in the 12 months preceding the survey, where the primary perpetrators were clients, followed by police and law enforcement officers, and finally, healthcare professionals [10]. Aiming towards accomplishing the UNAIDS goals established for 2030 [16], comprehending the factors linked to the reporting of stigma and violence is crucial, especially considering their impact on the prevalence of HIV. This study is important because it will provide evidence on how stigma, physical violence and sexual violence burden the population of FSW as well as identify the factors associated with these issues, offering crucial insights to guide decision-making as precisely as possible.

## Methods

### Study design

This study is a secondary analysis of data collected in the BBS carried out between September 2019 and 2020 among FSW of five urban areas (Maputo, Beira, Nampula, Zambezia, and Tete) in Mozambique. The primary aim of the original survey was to assess HIV prevalence and identify associated risk factors, understand healthcare access and estimate the size of the FSW population.

### Study population and sampling

The respondents of this survey included FSW aged 15 and above, with those under 18 considered emancipated minors, who were thereby eligible to provide written informed consent for participation. Eligibility criteria included FSWs who, in the six months prior to the survey, reported that they had received money for sexual services from someone other than their main partner and who resided, worked, or socialized in the survey areas. Additionally, possession of a valid referral coupon was a prerequisite for participation, in reference to the sampling methodology.

Given the high-risk nature of FSWs and the challenges in accessing this group through conventional sampling methods, respondent-driven sampling (RDS) was employed. This approach began with the identification and selection of initial participants (seeds), who then randomly selected three FSWs from their network. These new participants, in turn, selected three more each, continuing this process until the desired sample size was achieved. RDS is particularly effective for reaching hard-to-access populations, ensuring a more representative sample of the FSW community in the surveyed area. Research on the methodology has been described and published elsewhere [17–19].

### Data collection and analysis

A standardized questionnaire was used to collect data related to sociodemographic characteristics, sexual behaviors that could potentially increase the risk of HIV and other STIs, experiences of stigma and violence (both physical and sexual), symptoms and diagnoses of sexually transmitted infections (STIs), and access to healthcare services, including HIV testing. This questionnaire was then administered by pretrained enumerators using the ODK platform on mobile devices. These data were then cleaned and analyzed using the Respondent-Driven Sampling Analysis Tool (RDSAT) version 4.2.2 [20].

In this study, we focused on examining stigma, physical violence, and sexual violence among FSWs who participated in the survey. To facilitate this analysis, the responses to three key questions–each reflecting one of these aspects–were transformed into binary variables. This categorization created two distinct groups of FSWs: those who had experienced each type of violence (stigma, physical, or sexual) in the last 6 months before the survey and those who had not. Hazardous drinking was defined by the Alcohol Use Disorders Identification Test—Consumption (AUDIT-C) questionnaire. This tool is a brief alcohol screening instrument that helps identify individuals who are hazardous drinkers or have active alcohol use disorders. The AUDIT-C focuses on the frequency and quantity of alcohol consumption, categorizing drinking behaviors into various risk levels.

The data for this paper was accessed in May of 2023, and authors and investigators had no access to information that could identify the respondents. To analyze the association between each type of violence and the sociodemographic and behavioral characteristics, the chi-squared test was used, considering p<0.05.

Furthermore, to assess the effect of each associated variable and its magnitude on the likelihood of experiencing stigma, physical violence, or sexual violence, we conducted multivariate logistic regression analyses. This approach allowed us to control potential confounding variables and more accurately determine the impact of specific factors on the incidence of these types of violence among FSWs. Aditionally, we used backward stepwise variable selection procedure to remove less important variables from the multivariate logistic regression. The stepGAIC function from the Gamlss R package was utilized to perform the search of candidate variables for multivariate logistic regression, resulting in the selection of variables that minimized the Akaike’s information criterion (AIC) value [21].

### Ethical considerations

The study protocol was submitted and approved by both the Institutional and National Bioethics Committees for Health in Mozambique. Ethical clearance was specifically granted for the survey allowing the inclusion of FSW aged between 15 and 17 who were financially independent and residing away from their parents’ homes, therefore considered emancipated minors. Personal identifying data were not collected from the respondents to protect their privacy and ensure their safety, and all the included participants were previously informed about the purpose and risks of the survey and signed an informed consent form. All the staff involved in the survey were trained and signed a confidentiality agreement. All participants provided written informed consent for the behavioral questionnaire and HIV testing.

## Results

### Sociodemographic description of FSW participants

A total of 2,567 FSWs participated in the survey. Among the respondents, 39.5% were aged 25 years or older, 71.4% had attained a secondary level of education, and 92.2% were of Mozambican nationality. Additionally, 69.2% identified as Christian, 61.0% reported being single or never married, and 71.2% relied exclusively on sex work as their primary source of income during the six months preceding the survey. The main sociodemographic characteristics are summarized in Table 1.

**Table 1:** Unweighted pooled sociodemographic characteristics of FSW, 2019–2021.

Sociodemographic characteristics	N = 2567	% Adjusted (95% CI)
**Age range (year)**		
15–19	779	32.8 (30.1–35.6)
20–24	708	27.6 (25.7–29.5)
25 and above	1080	39.6 (36.8–42.3)
**Median age**	22 (19–30)	
**Level of education**		
No formal education/Primary	668	27.2 (25.5–28.9)
Secondary	1748	71.4 (69.8–73.1)
Superior	38	1.3 (1.2–1.5)
**Residence**		
Maputo	492	-
Beira	520	-
Tete	521	-
Quelimane	516	-
Nampula	518	-
**Nationality**		
Nacional	2341	92.2 (89.2–95.2)
Immigrant	224	7.8 (4.8–10.8)
**Religion**		
Christian	1800	69.7 (67.7–71.7)
Muslim	356	14.6 (12.7–16.5)
Other	406	15.7 (14.6–16.8)
**Marital status**		
Single/never married	1522	61.0 (58.7–63.3)
Married/cohabiting	183	6.8 (5.2–8.5)
Widowed/divorced/separated	859	32.2 (30.5–33.9)
**Had other job aside from sex work in** the 6 months prior to the survey	759	28.8 (26.6–30.9)

### Experience of stigma among FSW

Overall, 24,7% of the surveyed FSW reported experiencing stigma related to their occupation within the six months prior to the survey. Mozambican FSWs were more likely to experience stigma (25.3%, p<0.001), as were those who were married or cohabiting (25.0%, p<0.001), those who had engaged in work other than sex work in the 6 months prior to the survey (26.4%, p<0.001), and those who had a higher monthly income from sex work (over 100 USD) (26.8%, p<0.001). Risky behaviors associated with a higher report of stigma included having two or more fixed partners (25.8%, p = 0.031), having more than three clients in the month prior to the survey (p<0.001), meeting partners through the internet/SMS (27.9%, p<0.001) and through an agent (27.5%, p<0.001), performing sex work in the partner’s car or house (30.8%, p<0.001), using nonprescribed drugs (31.9%, p<0.001), having symptoms or a diagnosis of an STI in the six months prior to the survey (31.9%, p<0.001) and HIV-positive results (23.5%). These associations are summarized in Table 2.

**Table 2:** Weighted pooled sociodemographic and behavioral characteristics of FSW who experienced stigma, physical and sexual violence, Mozambique 2019–2020. P-values were obtained from chi-square tests, which were used to explore the associations between participants’ background variables and the outcome variables.

	Stigma			Physical violence			Sexual violence		
	n/N	% Adjusted (95% CI)	p value	n/N	% Adjusted (95% CI)	p value	n/N	% Adjusted (95% CI)	p value
**Age**									
15–19	197/779	24.0 (20.5–27.5)	0.065	398/779	51.2 (47.3–55.1)	0.002	302/779	36.3 (32.6–39.9)	0.106
20–24	193/707	25.0 (22.1–27.9)		381/707	53 (49.6–56.2)		287/707	38.4 (35.2–41.6)	
25+	243/1079	22.7 (20.5–24.9)		562/1079	52.5 (50.0–55.0)		386/1079	34.4 (32.1–36.8)	
**Level of education**									
Primary	159/668	23.1 (19.5–26.8)	0.071	347/668	52.7 (48.5–56.9)	0.072	228/668	32.5 (28.7–36.4)	<0.001
Secondary	450/1748	24.7 (22.3–27.1)		930/1748	52.9 (50.1–55.6)		705/1748	38.2 (35.6–40.9)	
Higher	14062	16.9 (5.5–28.3)		18/38	47.8 (30.9–64.7)		14/38	37.1 (21.4–52.8)	
**Nationality**									
Mozambican	619/2341	25.3 (23.3–27.4)	<0.001	1233/2341	52.1 (49.7–54.5)	<0.001	903/2341	36.4 (34.2–38.6)	0.015
Immigrant	14/224	5.5 (2.5–8.5)		108/224	53 (45.9–59.9)		72/224	32.8 (25.9–39.6)	
**Religion**									
Christian	464/1800	25.1 (22.7–27.6)	<0.001	969/1800	54.2 (51.5–56.8)	<0.001	684/1800	36.7 (34.1–39.2)	0.103
Muslim	81/356	21.2 (16.8–25.4)		153/356	42.5 (36.9–48.2)		140/356	35.8 (30.6–41.1)	
Other	88/406	20.3 (16.1–24.4)		218/406	52.4 (47.0–57.8)		151/406	34.3 (29.2–39.2)	
**Marital status**									
Single/never married	399/1522	24.4 (21.9–26.9)	0.023	790/1522	51.7 (48.9–54.6)	0.023	581/1522	35.9 (33.1–38.6)	0.044
Married/cohabiting	48/183	25 (18.6–31.5)		113/183	59.6 (51.9–67.3)		68/183	33.1 (25.8–40.3)	
Widowed/divorced/separated	186/859	22.4 (18.9–25.9)		438/859	51.5 (47.7–55.4)		326/859	37.2 (33.6–40.8)	
**Other work aside from sex work, last 6 months**	190/759	26.4 (22.7–30.0)	<0.001	409/759	55.9 (51.9–59.9)	<0.001	301/759	40.1 (36.1–43.9)	<0.001
**Monthly income from sex work**									
$55	180/809	20.4 (17.4–23.4)	<0.001	347/809	44.2 (40.5–48.0)	<0.001	283/809	33.6 (30.1–37.1)	
$55–100	201/858	23.5 (20.1–26.8)		473/858	54 (50.3–57.6)		332/858	37.2 (33.6–40.9)	<0.001
$100	221/825	26.8 (23.2–30.3)		468/825	57.3 (53.3–61.4)		341/825	38.7 (34.9–42.5)	
**Number of fixed sex partners (non-clients), last month**									
0	159/687	22.3 (18.6–25.9)	0.031	343/687	48.4 (44.2–52.5)	<0.001	248/687	35 (30.9–39.1)	<0.001
1	194/889	21.8 (18.6–24.9)		457/889	51.2 (47.4–55.0)		328/889	35 (31.5–38.5)	
2	241/893	25.8 (22.6–29.1)		483/893	55.4 (51.8–59.0)		373/893	38.8 (35.4–42.3)	
**Number of clients, last month**									
1–2	18/129	12.8 (6.9–18.8)	<0.001	55/129	48.2 (38.9–57.6)	<0.001	48/129	36.1 (27.6–44.7)	0.003
3–4	55/244	23.5 (17.4–29.7)		95/244	41.3 (34.4–48.1)		83/244	31.9 (25.6–38.3)	
5–6	57/244	22.5 (17.1–27.9)		118/244	47.2 (40.9–53.6)		96/244	37.1 (30.8–43.6)	
>7	503/1948	24.8 (22.6–26.8)		1073/1948	54.8 (53.1–56.6)		748/1948	36.7 (34.7–38.6)	
**Venue for meeting clients**									
Social establishments	13/155	7.3 (3.7–10.8)	<0.001	72/155	45.8 (37.4–54.2)	<0.001	56/155	33 (25.2–40.8)	0.016
Street/park	269/1196	23.6 (20.8–26.3)		629/1196	52.5 (49.4–55.7)		424/1171	36.6 (33.5–39.6)	
Agent	48/151	27.9 (20.7–35.2)		77/151	49.4 (40.7–58.2)		55/151	30.4 (22.9–37.9)	
Car/house of client of FSW	234/762	27.5 (23.9–31.1)		405/762	53.6 (49.5–57.7)		331/762	39.8 (35.8–43.8)	
Other	42/163	24.5 (18.1–31.1)		79/163	48.1 (40.2–56.0)		58/163	32.7 (25.8–39.6)	
**Venue for performing sex work**									
Social establishments	190/1022	17.7 (15.1–20.4)	<0.001	490/1022	47.7 (44.2–51.1)	0.499	330/1022	30 (26.9–33.2)	<0.001
Street/park	222/797	27.6 (24.1–31.1)		407/797	50.9 (46.9–54.9)		431/1196	36.4 (33.4–39.3)	
Car/house of client of FSW	175/553	30.8 (26.5–35.2)		338/553	61.5 (57.2–65.7)		229/553	40.4 (35.9–44.8)	
Other	40/150	25.4 (17.5–33.2)		81/150	54.6 (45.7–63.5)		74/150	48.6 (39.6–57.7)	
**Hazardous drinking** ^**a**^	366/1330	27 (24.2–29.7)	0.492	768/1330	58.3 (55.3–61.3)	<0.001	571/1330	41.1 (38.1–44.)	<0.001
**Illicit drug use, last 12 months**	120/342	31.9 (26.4–37.4)	<0.001	251/342	72.9 (67.8–78.0)	<0.001	194/342	54.2 (48.1–60.2)	<0.001
**Self-report STI, 12 months**	120/342	31.9 (26.4–37.4)	<0.001	485/776	64.4 (60.6–68.1)	<0.001	405/776	51.4 (47.4–55.4)	<0.001
**HIV positive result**	156/676	23.5 (20.6–26.4)	0.708	371/676	55.7 (52.4–58.9)	<0.0001	257/676	38.1 (34.9–41.2)	<0.001

^a^Hazardous drinking was defined by the Alcohol Use Disorders Identification Test—Consumption (AUDIT-C) questionnaire.

### Physical violence among FSW

Among the FSW who participated in the survey, over half (52.3%) reported experiencing physical violence related to their profession within the six months preceding the survey. Certain groups were more likely to report such episodes of physical violence. These included FSWs aged 20–24 years (53.3%, p = 0.002), those of foreign origin (53.0%, p<0.001), married or cohabiting individuals (59.6%, p = 0.023), those engaged in other forms of employment in addition to sex work (55.9%, p<0.001), and those earning more than $100 per month from sex work (57.3%, p<0.001).

Behavioral factors also influenced the likelihood of experiencing physical violence. Higher reports of physical violence were found among FSWs who had two or more sexual partners (non-clients) in the month before the survey (55.4%, p<0.001), those with seven or more clients (54.8%, p<0.001), those meeting clients through online/SMS platforms (53.6%, p<0.001), those with hazardous drinking behavior (58.3%, p<0.001), those who consumed unprescribed drugs (72.9%, p<0.001), and those who self-reported or were diagnosed with STIs (64.4%, p<0.001). Among FSW who experienced physical violence, 55.7% (p<0.001) were HIV positive (Table 2).

### Sexual violence among FSW

Among FSW participants, 37.9% reported experiencing sexual violence in the 6 months before the survey. A higher incidence of sexual violence was observed among FSWs with secondary education (38.2%, p<0.001), Mozambican nationals (36.4%, p = 0.015), and those who were widowed, separated, or divorced (37.2%, p = 0.044). FSWs engaged in additional work in addition to sex work (40.1%, p<0.001), and those earning more than $100 per month from sex work (38.7%, p<0.001) also reported higher levels of sexual violence (Table 2).

Regarding behavioral factors, sexual violence was higher among FSWs with two or more regular partners (38.8%, p<0.001), those with 5–6 clients per month (37.1%, p = 0.003), and those meeting clients online/SMS (39.8%, p = 0.016). Additionally, FSWs with hazardous drinking (41.1%, p<0.001), who used nonprescribed drugs (54.2%, p<0.001), who were diagnosed with or exhibited symptoms of STIs (51.4%, p<0.001) and who were HIV positive (38.1%, p<0.001) were among the groups with the highest reported rates of sexual violence (Table 2).

### Correlates of stigma, physical and sexual violence among FSW

When analyzing potential confounders in the multiple regression analysis, FSW with secondary education or higher were more likely to experience stigma (AOR = 1.13, p = 0.047). The likelihood of reporting stigma also increased with the number of clients in the month prior to the survey, with FSWs having more than seven clients (AOR = 6.11, p<0.001). Additionally, FSWs who conducted sex work in their clients’ cars or homes (AOR = 1.33, p<0.001), those with hazardous drinking habits (AOR = 1.33, p<0.001), and those who had symptoms or STI diagnosis in last year (AOR = 2.28, p<0.001) had greater odds of reporting stigma (Table 3).

**Table 3:** Weighted multivariate logistic regression of factors associated with stigma and physical and sexual violence among FSW Mozambique, 2019–2020.

	Stigma		Physical Violence		Sexual Violence	
	AOR (95%CI)	p value	AOR (95%CI)	p value	AOR (95%CI)	p value
**Age (Ref = 15–19)**						
20–24	1.10 (0.97–1.24)	0.125	1.06 (0.96–1.18)	0.257	1.06 (0.95–1.19)	0.28
25+	1.02 (0.90–1.15)	0.743	0.90 (0.79–1.03)	0.125	0.94 (0.83–1.08)	0.402
**Level of education (Ref = Primary)**						
Secondary/Higher	1.13 (1.00–1.27)	0.047	1.13 (1.02–1.24)	0.015	-	
**City (Ref = Maputo)**						
Beira	1.60 (1.38–1.85)	<0.001	2.12 (1.83–2.47)	<0.001	3.36 (2.85–3.95)	<0.001
Tete	0.23 (0.19–0.29)	<0.001	0.97 (0.84–1.12)	0.688	2.61 (2.20–3.09)	<0.001
Quelimanesupp	3.93 (3.35–4.61)	<0.001	1.96 (1.68–2.29)	<0.001	7.8 (6.58–9.24)	<0.001
Nampula	0.23 (0.19–0.29)	<0.001	0.55 (0.47–0.64)	<0.001	1.85 (1.56–2.17)	<0.001
**Nationality (Ref = Immigrant)**						
Mozambican	2.70 (1.96–3.72)	<0.001	-		1.69 (1.40–2.03)	<0.001
**Marital status (Re = Single/never married)**						
Married/cohabiting	-		1.49 (1.25–1.76)	<0.001	1.31 (1.09–1.57)	0.003
Widowed/divorced/separated	-		0.99 (0.90–1.09)	0.826	1.13 (1.02–1.25)	0.017
**Number of clients, last month (Ref = 1–2)**						
3–4	4.12 (2.99–5.65)	<0.001	0.95 (0.77–1.17)	0.629	0.92 (0.74–1.15)	0.475
5–6	4.09 (2.96–5.65)	<0.001	0.98 (0.79–1.21)	0.847	1.16 (0.93–1.46)	0.19
7+	6.11 (4.54–8.22)	<0.001	1.29 (1.07–1.55)	0.007	1.47 (1.21–1.78)	<0.001
**Venue for meeting clients (Ref = Social establishments)**						
Street/park	-		0.95 (0.8–1.13)	0.551	0.81 (0.67–0.97)	0.024
Agent	-		0.68 (0.55–0.86)	0.001	0.40 (0.31–0.51)	<0.001
Car/house of client of FSW	-		0.81 (0.68–0.97)	0.022	0.62 (0.51–0.76)	<0.001
**Venue for performing sex work (Ref = Social establishments)**						
Street/park	1.21 (1.07–1.37)	0.003	0.94 (0.85–1.04)	0.23	1.31 (1.18–1.47)	<0.001
Car/house of client of FSW	1.33 (1.17–1.52)	<0.001	1.51 (1.35–1.68)	<0.001	1.22 (1.09–1.37)	<0.001
**Monthly income from sex work (Ref = $55)**						
$55–100	1.17 (1.04–1.32)	0.01	1.55 (1.41–1.70)	<0.001	1.18 (1.07–1.31)	0.002
$100	1.47 (1.29–1.67)	<0.001	1.49 (1.34–1.65)	<0.001	1.18 (1.06–1.32)	0.003
**Hazardous drinking**	1.33 (1.21–1.48)	<0.001	1.17 (1.08–1.27)	<0.001	1.36 (1.24–1.48)	<0.001
**Illicit drug use, last 12 months**	-		2.29 (2.01–2.62)	<0.001	2.64 (2.32–2.99)	<0.001
**Self-report STI, last 12 months**	2.28 (2.07–2.52)	<0.001	1.50 (1.37–1.65)	<0.001	2.12 (1.94–2.33)	<0.001
**HIV positive result**	-		1.15 (1.04–1.28)	0.006	1.12 (1.01–1.25)	0.031

FSWs with a secondary education level or higher (AOR = 1.13, p = 0.015), married/cohabiting (AOR = 1.49, p<0.001), who performed sex work in clients’ homes or cars (AOR = 1.51, p<0.001), with hazardous drinking (AOR = 1.17, p<0.01), who used unprescribed drugs (AOR = 2.29, p<0.001) and those who had symptoms or a diagnosis of an STI (AOR = 1.50, p<0.001) in the past 6 months before the survey had greater odds of reporting physical violence (Table 3).

There were greater odds of reporting violence among Mozambican FSWs (AOR = 1.69, p<0.001), married or cohabiting (AOR = 1.31, p = 0.003), those with more than seven clients in the last month before the survey (AOR = 1.47, p<0.001), those who engaged in street/park-based sex work (AOR = 1.31, p<0.001), those with hazardous drinking (AOR = 1.36, p<0.001), those who reported unprescribed drug use in the six months before the survey (AOR = 2.64, p<0.001), and those experiencing symptoms or a diagnosed STI during the same period (AOR = 2.12, p<0.001). Notably, HIV-positive FSW face higher odds of experiencing both physical (AOR = 1.15, p = 0.006) and sexual violence (AOR = 1.31, p<0.001) when compared to their HIV-negative counterparts.

## Discussion

Our findings highlight significant levels of stigma, physical, and sexual violence among FSWs in Mozambique, with important implications for public health interventions. Stigma against FSWs remains a pervasive issue in the country, contributing to their vulnerability to HIV infection. Negative societal attitudes and discriminatory practices often restrict FSWs’ access to healthcare services and safer sexual practices [5, 9].There is a prevailing notion that FSW are perceived as immoral, indecent, disruptors of households, and potential vectors for diseases [9], and this negative connotation can significantly impact their healthcare seeking behavior, driven by the desire to avoid experiences of stigmatization, discrimination, and inadequate treatment [22, 23].

In our study, Stigma was notably higher among Mozambican FSWs compared to immigrant FSWs, likely due to the greater visibility of local FSWs within their communities, where they are recognized by family, friends, and acquaintances. In contrast, immigrant FSWs may face less scrutiny due to their outsider status. Societal attitudes often tend to tolerate outsiders’ behaviors while being more critical of similar behaviors among their own members [9]. Married or cohabiting FSWs also faced increased stigma, likely due to societal expectations that married women should not engage in sex work, especially considering their perceived roles as family role models [24].

FSWs who engaged in other jobs besides sex work reported higher stigma levels, possibly due to increased community interactions in these jobs, exposing them to more judgment. Those with higher incomes from sex work were also more stigmatized, likely due to the assumption that more clients and encounters increase visibility and exposure [9]. Alcohol consumption and nonprescribed drug use further intensified the stigma, as these behaviors are socially stigmatized and complicate access to health services [25]. Lastly, FSWs who self-reported an STI in the last 12 months experienced significantly increased stigma, as this may deter them from seeking medical care, exacerbating the cycle of stigma and risk [9].

In the context of sex work, violence often extends beyond the boundaries that separate different forms of violence and manifests collectively, leading to a convergence of physical, emotional, and sexual violence [14, 23]. The findings of this study reveal that over half of the FSW (52.3%) reported experiencing physical violence, and over one-third (37.9%) encountered sexual violence within the six months prior to the survey. Notably, clients, police, and friends commonly emerge as perpetrators in these incidents [14, 26].

Our study reveals that over half of the FSWs reported experiencing physical violence in the six months prior to the survey. Married or cohabiting FSW were found to have a higher likelihood of experiencing physical and sexual violence compared to their single or never married counterparts, contradicting previous research in Mozambique [14] where single FSWs were more frequently victimized. This difference could be attributed to domestic conflicts that arise when spouses discover their partner’s involvement in sex work, leading to violence due to feelings of shame or anger [14]. Furthermore, married FSWs may be more exposed and susceptible to both physical and sexual violence due to their lower likelihood of reporting abuse to avoid disclosing their engagement in sex work, thereby increasing their vulnerability [27]. Additionally, FSWs with a greater number of sexual partners, both regular and clients, reported higher rates of violence. Research from Mozambique and South Africa supports this trend, indicating an association between the number of partners and an increased risk of violence [14, 27]. Client-perpetrated violence often arises from disputes over sexual encounter negotiations or payment terms, and in the case of long-term relationships, it can be due to jealousy and possessiveness from the client [27]. Coercive and nonconsensual acts by clients, not agreed upon beforehand, are considered sexual violence [27].

Income derived from sex work also played a significant role in the risk of violence among FSW. Those with incomes exceeding $100 were at a higher risk of both physical and sexual violence compared to those with lower incomes. Higher incomes often indicate more clients and/or encounters, which may lead to demands for unsafe sexual practices, increasing the risks for FSWs [14]. Additionally, higher payments can limit their ability to negotiate safe practices during encounters, heightening vulnerability [10, 14]. Substance use, including unprescribed drugs and hazardous alcohol consumption, were associated with physical and sexual violence, particularly among women who consumed both substances. While substance use can serve as a coping mechanism within the sex work context, it reduces risk perception and impairs the ability to identify dangerous situations, ultimately increasing exposure to risky sexual behaviors and client aggression [9, 14, 27]. Moreover, the venue where FSWs meet their clients also significantly influences their risk of violence. Those who meet clients online or through SMS, as well as those who engage in encounters in street or park settings, face a heightened risk. Virtual interactions for client selection pose a risk, as they do not allow a pre-analysis of clients before the encounter [28], and street/park settings are usually associated with higher violence rates and are often located in areas with high alcohol consumption and elevated violence levels, creating an ideal scenario for physical and sexual aggression [10, 28].

Our study also revealed that higher levels of physical and sexual violence were reported by HIV-positive FSW (55.7% and 38.1%, respectively), and being HIV-positive was found to increase the chances of experiencing physical and sexual violence. Violence increases the risk of contracting HIV and other STIs. Studies show that women who experience violence are three times more likely to get HIV than those who do not [29]. This is because it hinders the negotiation of safe sexual conditions with clients, regular partners, or pimps, thereby increasing the risk of HIV infection [30]. The perpetrator of sexual violence is often characterized by no condom use, a high risk of HIV and other STIs, drug use, and aggressive action [4, 31]. This continuous cycle of violence prevents FSW from seeking justice services to report the assaults and accessing HIV prophylaxis healthcare services. Consequently, this directly impacts the growth of HIV cases and hinders the effectiveness of policies and interventions aimed at mitigating the epidemic’s growth.

Although our study provides valuable information about stigma and violence among FSW in Mozambique, some limitations need to be taken into account. First, the study lacks information about the perpetrators of violence, which makes it challenging to design tailored interventions. Second, the frequency of each type of violence is unspecified, making it difficult to distinguish between isolated incidents and recurrent patterns. Third, data on healthcare-seeking behavior among FSWs after experiencing violence are missing, which affects the assessment of service adequacy. Additionally, the study did not include high-class FSW, limiting our understanding of violence trends in higher socioeconomic contexts. Furthermore, the reliability of responses may be influenced by memory bias or discomfort in answering sensitive questions. Finally, it is important to note that the study’s findings may not be generalizable to other cities and provinces, underscoring the need for further research and a comprehensive approach to understanding violence against FSWS.

## Conclusion

Our study highlights the high prevalence of self-reported stigma and violence (both physical and sexual) among FSWs in Mozambique. Stigma, often driven by societal norms, hinders safe sexual practices and healthcare-seeking behaviors. The coexistence of physical and sexual violence is alarming, with factors such as income, number of sexual partners, client acquisition methods, substance use, and STI symptoms contributing to the heightened vulnerability of FSWs. Additionally, being HIV-positive is associated with an increased likelihood of experiencing both physical and sexual violence, further complicating the challenges faced by this marginalized group.

Considering Mozambique’s high HIV prevalence, it is crucial to enhance public health responses that specifically target high-risk groups like FSWs. Establishing legal protections for FSWs and improving access to justice through dedicated platforms for reporting violence and rights violations are essential. Public health initiatives must prioritize reducing stigma, promoting safer sexual practices, and improving access to healthcare, particularly for FSWs who are most vulnerable due to income, client acquisition methods, and substance use. Tailored, multi-faceted approaches are necessary to create a safer and more equitable environment for FSWs in Mozambique.

## References

[1] Richter ML, Chersich M, Temmerman M, Luchters S. Characteristics, sexual behaviour and risk factors of female, male and transgender sex workers in South Africa. South African Medical Journal. 2013;103: 246–251. doi: 10.7196/samj.6170 23547701

[2] UNAIDS. HIV and sex work—Human rights fact sheet series 2021. 2021 [cited 23 May 2022]. Available: https://www.unaids.org/en/resources/documents/2021/05-hiv-human-rights-factsheet-sex-work

[3] Ngugi EN, Roth E, Mastin T, Nderitu MG, Yasmin S. Female sex workers in Africa: Epidemiology overview, data gaps, ways forward. SAHARA J. 2012;9: 148–153. doi: 10.1080/17290376.2012.743825 23237069 PMC4560463

[4] Dunkle KL, Decker MR. Gender-Based Violence and HIV: Reviewing the Evidence for Links and Causal Pathways in the General Population and High-risk Groups. American Journal of Reproductive Immunology. 2013;69: 20–26. doi: 10.1111/aji.12039 23216606

[5] UNAIDS. Evidências para eliminar estigma e discriminação relacionados ao HIV. Geneva; 2020.

[6] Ma H, Loke AY. A qualitative study into female sex workers’ experience of stigma in the health care setting in Hong Kong. International Journal for Equity in Health. 2019;18: 175. doi: 10.1186/s12939-019-1084-1 31727157 PMC6857210

[7] Durisin EM, Van der Meulen E, Bruckert C. Red Light Labour: Sex Work Regulation, Agency, and Resistance. Vancouver: UBC Press. 2018.

[8] Holzemer WL, Uys LR, Chirwa ML, Greeff M, Makoae LN, Kohi TW, et al. Validation of the HIV/AIDS Stigma Instrument—PLWA (HASI-P). AIDS Care. 2007;19: 1002–1012. doi: 10.1080/09540120701245999 17851997

[9] Nguyen Thi Van Anh VTTN (Mit) Pham Duc Cuong. UNDERSTANDING AND CHALLENGING STIGMA TOWARD SEX WORKERS AND HIV IN VIETNAM. 2011. Available: https://www.icrw.org/wp-content/uploads/toolkit-for-action.pdf

[10] Fonds Aids. Trabalho de sexo e violência em Moçambique. Relatório de avaliação de necessidades. 2016.

[11] Marks G, Crepaz N, Janssen R. Estimating sexual transmission of HIV from persons aware and unaware that they are infected with the virus in the USA. AIDS (London, England). 2006;20: 1447–50. doi: 10.1097/01.aids.0000233579.79714.8d 16791020

[12] Ruegsegger LM, Stockton M, Go VF, Piscalko H, Davis D, Hoffman IF, et al. Stigma, Social Support, and Sexual Behavior among Female Sex Workers at risk for HIV in Malawi. AIDS Educ Prev. 2021;33: 290–302. doi: 10.1521/aeap.2021.33.4.290 34370569 PMC8408828

[13] African Sex Worker Aliance. Violence against Sex Workers in Africa–ASWA Alliance Africa. 2019 [cited 9 Mar 2023]. Available: https://aswaalliance.org/download/violence-against-sex-workers-in-africa/

[14] Ngale K, Cummings B, Horth R. Unseen, unheard and unprotected: prevalence and correlates of violence among female sex workers in Mozambique. Cult Health Sex. 2019;21: 898–913. doi: 10.1080/13691058.2018.1524512 30451098 PMC11669849

[15] AIDSInfo UNAIDS. Global data on HIV epidemiology and response. 2022 [cited 9 Mar 2023]. Available: https://aidsinfo.unaids.org/

[16] UNAIDS. Fast-Track strategy to end the AIDS epidemic by 2030. 2016 [cited 3 Nov 2023]. Available: https://www.unaids.org/en/resources/campaigns/World-AIDS-Day-Report-2014

[17] Heckathorn D. Respondent-Driven Sampling II: Deriving Valid Population Estimates From Chain-Referral Samples of Hidden Populations. Social problems. 2002;49: 11–34. doi: 10.1525/sp.2002.49.1.11

[18] Semá Baltazar C, Boothe M, Chitsondzo Langa D, Sathane I, Horth R, Young P, et al. Recognizing the hidden: strengthening the HIV surveillance system among key and priority populations in Mozambique. BMC Public Health. 2021;21: 91. doi: 10.1186/s12889-020-10110-y 33413261 PMC7789885

[19] Augusto Â do R, Young PW, Horth RZ, Inguane C, Sathane I, Ngale K, et al. High burden of HIV infection and risk behaviors among female sex workers in three main urban areas of Mozambique. AIDS Behav. 2016;20: 799–810. doi: 10.1007/s10461-015-1140-9 26238035 PMC5092171

[20] Team R. A language and environment for statistical computing. Computing. 2006;1. doi: 10.1890/0012-9658(2002)083[3097:CFHIWS]2.0.CO;2

[21] Rigby RA, Stasinopoulos DM. Generalized additive models for location, scale and shape. Journal of the Royal Statistical Society Series C. 2005;54: 507–554.

[22] Bitty-Anderson AM, Gbeasor-Komlanvi FA, Tchankoni MK, Sadio A, Salou M, Coffie PA, et al. HIV prevalence and risk behaviors among female sex workers in Togo in 2017: a cross-sectional national study. Archives of Public Health. 2022;80: 92. doi: 10.1186/s13690-022-00851-0 35331303 PMC8943989

[23] Mabuie M. Comunicação em saúde: evidências e desafios na prevenção do VIH em mulheres trabalhadoras de sexo na cidade de Maputo, Moçambique. Religación: Revista de Ciencias Sociales y Humanidades. 2021;6: 1.

[24] Gois ML de, Lima MEO. De dentro de fora e de fora de dentro: representações sociais da prostituição feminina. Interacções. 2013;9. doi: 10.25755/int.2820

[25] Benoit C, McCarthy B, Jansson M. Stigma, sex work, and substance use: a comparative analysis. Sociology of Health & Illness. 2015;37: 437–451. doi: 10.1111/1467-9566.12201 25688450

[26] INS C UCSF, Pathfinder International, I-tech. Relatório Final: Inquérito Biológico e Comportamental entre Mulheres Trabalhadoras de Sexo em Moçambique, 2011–2012. São Francisco: UCSF; 2013.

[27] Jewkes R, Otwombe K, Dunkle K, Milovanovic M, Hlongwane K, Jaffer M, et al. Sexual IPV and non-partner rape of female sex workers: Findings of a cross-sectional community-centric national study in South Africa. SSM Ment Health. 2021;1: None. doi: 10.1016/j.ssmmh.2021.100012 34957423 PMC8654680

[28] Amogne MD, Balcha TT, Agardh A. Prevalence and correlates of physical violence and rape among female sex workers in Ethiopia: a cross-sectional study with respondent-driven sampling from 11 major towns. BMJ Open. 2019;9: e028247. doi: 10.1136/bmjopen-2018-028247 31366648 PMC6678027

[29] Prakash R, Manthri S, Tayyaba S, Joy A, Raj SS, Singh D, et al. Effect of Physical Violence on Sexually Transmitted Infections and Treatment Seeking Behaviour among Female Sex Workers in Thane District, Maharashtra, India. PLoS One. 2016;11: e0150347. doi: 10.1371/journal.pone.0150347 26933884 PMC4774990

[30] Decker MR, Patel SA, Srikrishnan AK, Celentano DD, Solomon S, Sherman SG. Condom nonuse among female sex workers in Chennai, India. Int J Gynaecol Obstet. 2011;114: 156–157. doi: 10.1016/j.ijgo.2011.02.017 21669420 PMC3133810

[31] Beksinska A, Prakash R, Isac S, Mohan HL, Platt L, Blanchard J, et al. Violence experience by perpetrator and associations with HIV/STI risk and infection: a cross-sectional study among female sex workers in Karnataka, south India. BMJ Open. 2018;8: e021389. doi: 10.1136/bmjopen-2017-021389 30206080 PMC6144389

